# Poly ADP-Ribosylation in a Plant Pathogenic Oomycete *Phytophthora infestans*: A Key Controller of Growth and Host Plant Colonisation

**DOI:** 10.3390/jof11010029

**Published:** 2025-01-03

**Authors:** Viktoriya O. Samarskaya, Sofya Koblova, Tatiana Suprunova, Eugene A. Rogozhin, Nadezhda Spechenkova, Sofiya Yakunina, Andrew J. Love, Natalia O. Kalinina, Michael Taliansky

**Affiliations:** 1 Shemyakin-Ovchinnikov Institute of Bioorganic Chemistry of the Russian Academy of Sciences, 117997 Moscow, Russia; v.sam@ibch.ru (V.O.S.); rea21@list.ru (E.A.R.);; 2 Doka-Gene Technologies Ltd., Moscow Region, 141880 Rogachevo, Russia; koblovasofya521@gmail.com (S.K.); suprunova@gmail.com (T.S.); 3 James Hutton Institute, Invergowrie, Dundee DD2 5DA, UK; andrew.love@hutton.ac.uk; 4 Belozersky Institute of Physico-Chemical Biology, Lomonosov Moscow State University, 119991 Moscow, Russia; kalinina@belozersky.msu.ru

**Keywords:** *Phytophthora infestans*, potato, RNA interference, dsRNA, poly ADP-ribosylation, PARP inhibitor

## Abstract

ADP-ribosylation is a reversible modification of proteins and nucleic acids, which controls major cellular processes, including DNA damage repair, cell proliferation and differentiation, metabolism, stress, and immunity in plants and animals. The involvement of ADP-ribosylation in the life cycle of *Dictyostelium* and some filamentous fungi has also been demonstrated. However, the role of this process in pathogenic oomycetes has never been addressed. Here, we show that the *Phytophthora infestans* genome contains two PARP-like protein genes (PiPARP1 and PiPARP2), and provide evidence of PARylation activity for one of them (PiPARP2). Using dsRNA-mediated RNA silencing of the PiPARP2 gene and chemical (pharmacological) inhibition of PARP activity by 3-aminobenzamide (3AB) PARP inhibitor, we demonstrate the critical functional role of ADP-ribosylation in *Phytophthora* mycelium growth. Virulence test on detached leaves also suggests an important role of ADP-ribosylation in *Phytophthora* host plant colonisation and pathogenesis. On a practical level, our data suggest that targeting the PARylation system may constitute a novel powerful approach for the management of *Phytophthora* diseases.

## 1. Introduction

Late blight of potatoes and tomatoes is caused by the oomycete *Phytophthora infestans* (Mont.) de Bary, which is one of the most harmful plant pathogens that annually leads to substantial crop yield losses [1]. The establishment of this disease is governed at the molecular level by the biosynthesis of numerous effector proteins by the pathogen and the expression of avirulence genes by the host [2]. These effectors directly interact with components of the plant’s immune system, determining the level of pathogenesis and degree of aggressiveness. As the resistance of *P. infestans* to chemical fungicides becomes increasingly prevalent, there is a need to establish more effective alternative environmentally friendly methods of its control based on new knowledge of its biology and interaction with its host.

ADP-ribosylation is a system that modifies target molecules including proteins, RNA and DNA via the transfer of ADP-ribose moieties from nicotinamide adenine dinucleotide (NAD+) either in the form of ramifying or linear polymers of ADP-ribose (PARylation), or a single ADP-ribose unit (MARylation) [3,4,5,6,7,8,9]. ADP-ribosylation is catalysed by poly(ADP-ribosyl) polymerases (PARPs), also known as ADP ribosyltransferases (ARTs) [6]. Typically, the resultant PARylated molecules, including PARP itself, function as regulators of various biological processes. During these processes PAR chains on target molecules could be hydrolysed by poly(ADP-ribose) glycohydrolases (PARGs), which results in the release of ADP-ribose or free PAR [3,4,5,6,7,8,9]. Subsequently, ADP-ribose or free PAR are cleaved into AMP and ribose-5-phosphate via the activity of nucleoside diphosphate linked to some moiety-X (NUDIX) hydrolases [10].

**Figure 1:**
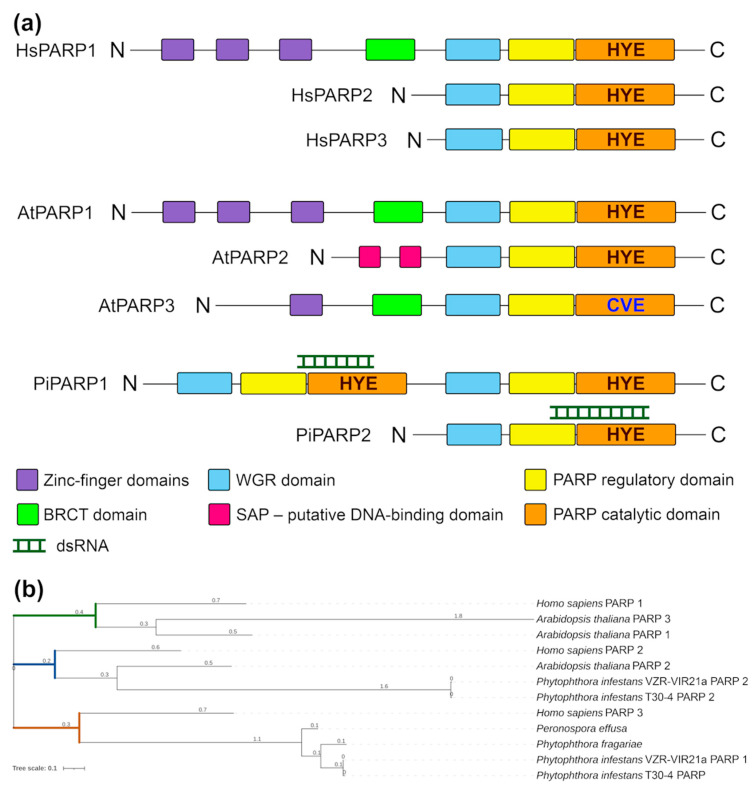
Domain structure and phylogenetic analysis of PARP proteins. (**a**) Schematic architecture of domains present in PARP proteins and determined using Pfam 27.0. Protein domains are shown as coloured boxes. Classical His-Tyr-Glu (HYE) triad in the CAT domain is replaced by a Cys-Val-Glu (CVE) motif in AtPARP3 as shown. (**b**) Maximum-likelihood phylogenetic tree of PARPs identified in *Homo sapiens*, *Arabidopsis thaliana*, *Phytophthora infestans*, *Phytophthora fragariae* and *Perenospora effuse* (three latter species belong to the clade *Stramenopiles*). This tree is based on a multiple alignment that includes all PARP domains.

Signs of ADP-ribosylation are observed in all kingdoms of life. Accordingly, PARP proteins and PARP homologs have been discovered or predicted in animals, plants, fungi, bacteria and viruses [6]. In the human genome, 17 PARP gene members (HsPARPs) have been recognised. They encode a group of proteins with distinct structural domains, functions and intracellular localisations [11]. HsPARP1, the founding member of the PARP family in humans has six main functional domains arranged in a modular manner from the N to the C terminus [12] (Figure 1a). At the N-terminal region, three zinc-finger DNA-binding domains (Zn1, Zn2, and Zn3) are responsible for detecting DNA breaks, which abuts a distal breast cancer type 1 susceptibility protein (BRCA1) C-terminal (BRCT) domain that mediates protein–protein binding and contains the site for PARP1 auto-modification. The C-terminal to the BRCT module is the WGR domain, which has the conserved Trp-Gly-Arg motif that is involved in interactions with DNA and controls the catalytic activity of PARP1. The C-terminal end consists of a catalytic domain (CAT), which contains a “PARP signature” comprised of the auto-inhibitory helical subdomain (PARP regulatory domain, REG) and a bona fide ADP-ribosylation subdomain (PARP CAT domain) [12]. This is in contrast to HsPARP1, HsPARP2 (also PARylation protein), and HsPARP3 (MARylation protein), which have more compact architectures with shorter N-terminal extensions from the WGR domain that completely lack zinc-finger DNA-binding domains [13]. Common to most CAT domains is the triad motif His-Tyr-Glu, which is essential for the catalytic activity of PARP1 [3]. In addition to HsPARP1, HsPARP2 and HsPARP3 (which are activated upon DNA damage), other members of the PARP family are structurally similar and described in human cells including tankyrases (PARP5a, PARP5b), which are involved in telomere homeostasis and cellular signalling, macrodomain-containing PARPs (macroPARPs, HsPARP9, HsPAR14 and HsPARP15) and others, which are still largely uncharacterised in terms of their functions [5].

The plant (*Arabidopsis* spp.) genomes encode three PARP genes: PARP1, PARP2 and PARP3. The PARP1 protein from Arabidopsis (AtPARP1), has conserved domain organisation that is quite similar to HsPARP1 (Figure 1a). AtPARP1 contains three zinc-finger domains, a BRCT domain, a WGR domain, a PARP regulatory (REG) and a PARP catalytic (CAT) domain [5,14] (Figure 1a). The domain architecture of AtPARP2 differs from that of PARP1 and resembles HsPARP2 and HsPARP3 (Figure 1a) [5,14]. However, in addition to the WGR domain, REG domain and CAT domain, AtPARP2 contains two putative DNA-binding SAP domains in the N-terminal region (Figure 1a), named after three proteins that contain it (SAF-A/B, Acinus and PIAS) [5,14]. AtPARP3 contains one zinc-finger domain, BRCT domain, WGR domain, PARP REG and PARP CAT domains [5,14,15] (Figure 1a). Interestingly, the classical functional triad His-Tyr-Glu in the CAT domain of AtPARP3 is replaced by a Cys-Val-Glu motif. Moreover, AtPARP3 is missing poly ADP-ribosylation activity in plants and apparently performs functions different from those of AtPARP1 and AtPARP2 [15].

In animals and plants, ADP-ribosylation operates in order to regulate a broad range of cellular functions including DNA repair, gene expression, chromatin remodelling and programmed cell death [5,6,14,16]. In plants, PARP proteins play a key role in the regulation of immunity and abiotic stress tolerance [7,8,17]. Involvement of PARylation process in the life cycle of unicellular eukaryote, *Dictyostelium* spp. [18] and filamentous fungi, such as *Aspergillus nidulans* [19], *Neurospora crassa* [20], *Fusarium oxysporum* [21] or *Magnaporthe oryzae* [22], have also been demonstrated.

In contrast, to the best of our knowledge, the role of PARylation in oomycete pathogens has not been addressed elsewhere. Here, we show that the *P. infestans* genome contains two PARP-like protein genes (PiPARP1 and PiPARP2) and provide evidence of PARylation activity for one of these proteins (PiPARP2). Using dsRNA-mediated RNA interference and chemical inhibition of PARP activity PARP inhibitor, we demonstrate the critical functional role of ADP-ribosylation in *Phytophthora* mycelium growth. Virulence test on detached leaves also suggests an important role of ADP-ribosylation in *Phytophthora* host plant colonisation and pathogenesis.

## 2. Materials and Methods

### 2.1. DNA Extraction, Purification and Sequencing

*Phytophthora infestans* VZR ViR21a strain (with A1 mating type and virulence race R 1, 2, 3, 4, 5, 6, 7, 8, 10, 11) was collected in the Bryansk region of Russia in 2021, and provided by the All-Russian Institute of Plant Protection (FSBSI VIZR, Saint-Petersburg-Pushkin, Russia). DNA extraction was performed essentially as described by [23]. VZR ViR21a mycelial cultures were grown on potato sucrose agar (PSA) in the dark for 7–10 days. Consequently, mycelium, collected with a microbiological loop, was homogenised in a lysis buffer (1 M Tris-HCl, pH 7.5; 5 M NaCl; 0.5 M EDTA, pH 8.0; 2% cetyltrimethylammonium bromide (CTAB); 1% mercaptoethanol) preheated to 65 °C (100 mg of mycelium: 5 mL of buffer). The homogenate was incubated at 65 °C for 1.5 h (h), vortexing periodically. Then, an equal volume of chloroform was added, mixed and centrifuged at 5000 rpm for 10 min. The upper phase was transferred to a new tube and an equal volume of isopropanol was added, vortexed and incubated at −20 °C for 30 min. After centrifugation at 5000 rpm for 20 min, the pellet was washed with 70% ethanol and dissolved in 500 μL of Milli Q water. Then, 1 μL RNase A (10 mg/mL) was added. After incubation at 37 °C for 30 min, equal volumes (500 μL) of phenol and chloroform were added to the sample. The samples were centrifuged at 13,000 rpm for 5 min, and after that, the upper phase containing the DNA was transferred to new tubes and precipitated with 96% ethanol in the presence of 1/10 volume of 3 M sodium acetate. Finally, centrifugation was performed, the supernatant was removed and the pellet was dissolved in 100 μL of Milli Q water. The DNA quantity and quality were determined using a NanoDrop spectrophotometer (Thermo Fisher Scientific, Waltham, MA, USA). The PacBio sequencing and demultiplexing of the HiFi reads was performed by CeGaT GmbH (Tuebingen, Germany). The genome of *P. infestans* strain VZR ViR21a was assembled from HiFi reads using Hifiasm (v.0.19.8) with default settings, with a resulting N50 over 7 Mbp and BUSCO C: 99.0% [S: 89.0%, D: 10.0%], F: 0.0%, M: 1.0%, n: 100 (dataset: stramenopiles_odb10) and the genome assembly size was 222.34 Mbp. The homology of the amino acid sequences between PARP proteins of *P. infestans* VZR ViR21a, *P. infestans* T30-4, *Homo sapiens* and *Arabidopsis thaliana* was evaluated by constructing a phylogenetic tree using the Maximum Likelihood method and the Whelan and Goldman model within MEGA11 software v.11.0.10 [24] with 1000 bootstrap replicates to determine statistical significance. The trees were visualised using iTOL v.6.8 [25].

### 2.2. Production and Purification of dsRNA

To design dsRNA constructs able to specifically silence *P. infestans* VZR ViR21a genes, we selected the conservative fragments of the PiPARP1 CDS with a length of 662 bp (nts 1594–2255), PiPARP2 CDS with a length of 813 bp (nts 1225–2037) and PiPARG CDS with a length of 698 bp (nts 1–680) extracted from the whole genome sequence of VZR ViR21a (Supplementary Figure S1). In order to select the regions of *PiPARP1*, *PiPARP2* and *PiPARG* genes as targets by dsRNAs, we first used the E-RNAi tool (http://e-rnai.dkfz.de/; accessed on 20 December 2022), which is widely used for the identification of RNAi target sites by ranking sequences according to their predicted specificity, efficiency and complexity [26]. Then, to avoid off-target silencing, all these fragments were screened using the siRNA scan website (http://bioinfo2.noble.org/RNAiScan.htm; accessed on 20 December 2022); to identify 21 nt stretches that have homology with other genes of *Phytophthora* and, thus, have the potential for “off-target” silencing. No sequence matches were found in any of the fragments in the genome of *P. infestans*. Finally, using preliminary tests on *P. infestans* mycelium, we confirmed the high efficiency and specificity of RNAi induced by selected dsRNAs on the genes of interest. It should be noted that the resulting PiPARP1 dsRNA was able to specifically target one of the duplicated “signature” PARP modules comprised of the parts of REG and CAT subdomains (which is located in the N-terminal part of the gene; Figure 1a); PiPARP2 dsRNA also targets the REG-CAT module, which is, however, not duplicated in the PiPARP2 gene (Figure 1a).

cDNA constructs corresponding to selected fragments were synthesised by Evrogen (Moscow, Russia) and cloned into the plasmid vector L4440 (plasmid 1654; Addgene Watertown, MA, USA). This vector contains two T7 promoters in an inverted orientation that flanks the multiple cloning sites. cDNA corresponding to the conservative fragment of the PVY replicase gene, which has a length of 500 bp (nts 7739–8238; Gene Bank accession number OR545670) was produced earlier [27,28]. To amplify the required fragment, the primers for the PCR reaction were designed with the T7 promoter sequence added to one of the primers. Two separate PCR reactions were performed to obtain the different PCR products with the T7 promoter of the different ends of the original sequence. The synthesised PCR products were used as templates for in vitro transcription of RNA using the Biolabmix mRNA-20 Synthesis Kit (Novosibirsk, Russia) following the manufacturer’s protocol. To form the dsRNA duplexes, equal volumes of the corresponding complementary ssRNAs were combined. The annealing buffer was added to the mixture up to the final concentration of 10 mM Tris-HCl pH 7.5, 2.5 mM MgCl_2_ and 0.1 mM CaCl_2_. Then, the mixture was annealed at 95 °C in a thermostat for 10 min and gradually cooled down to room temperature (over a period of no less than 90 min). The dsRNA was quantified using a NanoDrop spectrophotometer and examined in 1.2% agarose gels.

### 2.3. Treatment of Mycelium with dsRNA and PARP Inhibitor 3-Aminobenzamide (3AB)

*P. infestans* inoculum was prepared from 7-day-old cultures grown on rye agar medium in the dark at 18 °C. A bit of agar was placed in the centre of the new rye agar plate on the drop of dsRNA (150 ng/mL) or 3-aminobenzamide (3AB) (1 mM) solution (Sigma-Aldrich; St. Louis, MO, USA). Plates were incubated under 18 °C for 10 days to observe the growth of mycelium in the presence of dsRNAs, 3AB and controls [pure water for dsRNAs and 0.06% dimethyl sulfoxide (DMSO) for 3AB].

### 2.4. Real-Time Quantitative RT-PCR (RT-qPCR)

An amount of 500 mg of fresh mycelium was ground to a fine powder in liquid nitrogen using a pestle and mortar, and RNA was isolated using TRIzol as described in [29]. Aliquots of the treated RNA were used in transcription reactions (following the SuperScriptTM First-strand Synthesis System for RT-PCR (Invitrogen, Waltham, MA, USA), to produce cDNA. The primer pairs for SYBR green-based real-time PCR analysis of PiPARP1, PiPARP2 and PiPARG expression (designed using PRIMER EXPRESS software v3.0.1; Applied Biosystems, Foster City, CA, USA) were located outside of the fragments used for dsRNA construction to allow for the measurement of gene expression, rather than dsRNA accumulation, and are listed in Supplementary Table S1. Concentrations of primers with the lowest threshold cycle (Ct) values were used in the subsequent analysis in conjunction with 10-fold dilutions in sterile water of the first-strand cDNA reaction mixes. These components were combined with reagents in the QuantiTectTM SYBR^®^ Green PCR kit (Qiagen; Crawley, UK) according to the manufacturer’s instructions, prior to placing it in an ABI PRISM 7700 (Applied Biosystems, Foster City, CA, USA). Sequence Detection System for the amplification of products, using the following reaction conditions: 95 °C for 15 min, followed by 40 cycles of 94 °C for 15 s, 60 °C for 30 s and 72 °C for 30 s. The Ct value for each gene was normalised to reference gene mRNAs for ef1a elongation factor and 40S ribosomal protein S3a (Supplementary Table S1).

### 2.5. Immunological Detection of Poly ADP-Ribose (PAR)

Total protein for mycelium was isolated using simultaneous precipitation and denaturation with 2-mercaptoethanol (2ME) and trichloracetic acid (TCA) in cold acetone [30]. The protein was analysed for PAR accumulation levels by ELISA using LysA™ Universal PARylation Assay Kit (BPS Bioscience, San Diego, CA, USA).

### 2.6. Test on Detached Leaves

For the evaluation of disease development on detached leaves *P. infestans,* mycelia were harvested in sterile water and mixed with or without 3AB (1 mM) and stimulated to release zoospores at 4 °C for 3 h. The sporangia suspension was observed under a microscope and the concentration of zoospores was adjusted to 4 × 10^4^/mL for use as an inoculum. To infect potato leaves, zoospore suspension was drop-inoculated on leaves from the 40-day-old plant’s cv Gala and placed in a dark incubator at 16 °C, and the lesions were scored at 4 and 7 days post-inoculation (dpi). The leaves of potato plants cv. Gala were placed abaxial side up inside the plastic trays lined with wet paper and challenge inoculated with *P. infestans* zoospore suspension mixed with or without 3AB. The lesion area (LA) was measured using ImageJ software (Version 1.51) [31].

## 3. Results

Recently, using the PacBio platform, we sequenced the *P. infestans* strain VZR ViR21a genome (BioSample accession SAMN434845453), which was assembled from HiFi reads and was used as a reference sequence for mapping the coding sequences (CDS). PARP-associated sequences were identified using the Gene Ontology (GO) Term (GO tag GO:0003950), along with BLAST and HMMER searches against a range of known PARPs among human and plant genomes; two *P. infestans* (strain T30-4) coding sequences were found, which were previously annotated but not experimentally tested as putative PARPs, namely, PITG_17599 and PITG_12516, were also used for PARP gene mapping and assembly. Two coding sequences Vir21a1 and Vir21a2 (Supplementary Figure S2) extracted from the whole genome sequence of VZR ViR21a displayed a high level of similarity to PITG_17599 and PITG_12516, respectively. In total, Vir21a1 and Vir21a2 had 19 and 5 SNPs respectively, compared to strain T30-4.

To investigate the domain structure of these putative PARP proteins, we searched for all known PARP domains (Figure 1a) taken from the annotation of the InterPro database, which mapped to Vir21a1 CDS (hereinafter referred to as PiPARP1) and Vir21a2 CDS (hereinafter referred to as PiPARP2) using blastx. Both the putative PARPs had three domains: WGR, REG and CAT (Figure 1a). Phylogenetic analysis showed that PiPARP2 exhibited close relatedness to its orthologues in *A. thaliana* (AtPARP2) and *H. sapiens* (HsPARP2) (Figure 1b). However, in contrast to PiPARP2, PiPARP1 displayed major domain duplication (a feature found only in the clade *Stramenopiles*) of all the WGR, REG and CAT domains located at the N-terminus but fully duplicated at the C-terminus (Figure 1a). Interestingly, despite domain duplication, phylogenetic analysis suggested that PiPARP1 tends to be more closely related to HsPARP3 (Figure 1b).

Using similar approaches, we identified a coding sequence for the PARG gene in the VZR ViR21a strain of *P. infestans*, which presents as the single gene PiPARG (Supplementary Figure S2). The mRNA of the VZR ViR21a PiPARG sequence has 99% homology with PARG from the T30-4 strain (PITG_10553). In total, the PARG sequence from the VZR ViR21a strain had 3 SNPs—382A>G, 446C>T and 677 A>G—compared with the T30-4 strain.

Next, to obtain evidence on the role and biological function of ADP-ribosylation in *P. infestans*, we applied genetic and pharmacological approaches. As a genetic assay, we exploited an RNA interference (RNAi) method based on the use of externally delivered dsRNA [32] designed to target the mRNAs of PiPARP1, PiPARP2 and PiPARG (Supplementary Figure S1). This RNAi-based approach has been proven to be a robust, fast and efficient method that is widely employed in the discovery of gene functions in all kingdoms of life [33]. In the pharmacological approach, we used 3-aminobenzamide (3AB) as the chemical inhibitor blocking PARP (PARylation) activity [34].

**Figure 2:**
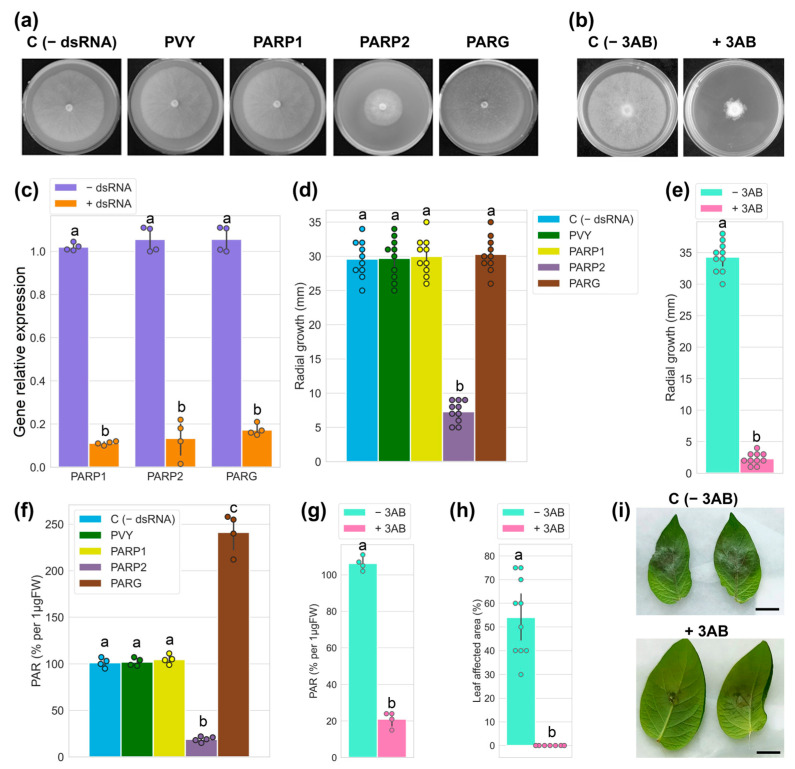
Effect of poly ADP-ribosylation on *P. infestans* growth and host plant colonisation. (**a**) Genetic (RNAi) assays to analyse effects of dsRNA corresponding to PiPARP1 (PARP1), PiPARP2 (PARP2) and PiPARG (PARG) (150 ng/µL) compared with controls [-dsRNA and non-specific potato virus Y dsRNA (PVY)]. (**b**) Pharmacological approach to analyse effect of 3-aminobenzamide (3AB) (1 mM) on *P. infestans* mycelial growth. (**a**,**b**) Photographs and (**d**,**e**) radial growth of 10-day-old colonies). (**c**) Effect of PiPARP1 (PARP1), PiPARP2 (PARP2) and PiPARG (PARG) dsRNAs on expression of the corresponding genes compared with control (-dsRNA) 10 days after treatment. (**f**,**g**) Accumulation of PARylated proteins measured by ELISA (expressed as percentage per 1 µgFW) in the presence or absence of dsRNA (**f**) or 3AB (**g**). (**h**,**i**) Development of symptoms 4 days post-inoculation (**i**) in the presence or absence of 3AB; lesion areas (**h**) were measured using ImageJ software. Statistical analysis was performed on ten (**d**,**e**,**h**) and four (**c**,**f**,**g**) independent biological replicates using Tukey’s HSD post hoc test and ANOVA. The different letters (a,b,c) indicate significant differences in mycelial diameter, PARP and PAR accumulation levels and leaf lesions.

In the first (RNAi) approach, drops of dsRNAs (150 ng/mL) were placed on rye agar medium in the centres of the plates, and, subsequently, inverted agar slices of *P. infestans* mycelium were added on top of the dsRNA. Plates were incubated under 18 °C for ten days and the growth of mycelium was monitored in the presence of the specific (PiPARP1, PiPARP2 and PiPARG dsRNAs) and non-specific (designed to target potato virus Y, PVY and used as a control) dsRNAs [28] (Figure 2a). Water plated medium was used as an additional control for *Phytophthora* growth (Figure 2a). In the second (pharmacological) approach, instead of dsRNAs, we used drops of PARP inhibitor 3AB (Figure 2b).

The expression levels of all mRNAs (PiPARP1, PiPAR2 and PiPARG) were suppressed approximately 10–15-fold in all cases by homologous dsRNA when compared with a control containing no dsRNA (Figure 2c). However, the inhibition of colony growth after the administration of dsRNA was observed only in the case of PiPARP2 dsRNA (Figure 2a,d). Silencing specificity induced by PiPARP2 dsRNA and its direct cross-talk with mycelial growth is supported by the data that show no effects of PiPARP1 (which, presumably, is not involved in these processes) dsRNA, PARG dsRNA and another non-specific dsRNA (PVY) on mycelial growth compared with the water control (Figure 2a,d). Interestingly, the PARP inhibitor 3AB also strongly suppressed the growth of *P. infestans mycelium* (Figure 2b,e). Collectively, these data suggested that the effects of PiPARP2 and 3AB may be attributed to PARylation rates. To confirm this idea, we detected a significant reduction in PAR levels in *P. infestans* mycelium treated with PiPARP2 dsRNA (Figure 2f) or 3AB (Figure 2g), which was accompanied by the inhibition of mycelial growth. A possibility of direct cross-talk between mycelial growth and PARylation rates is supported by the data that show a correlation between PAR accumulation levels and mycelial growth (Figure 2a,b,d–g). It should also be noted that PiPARG-dsRNA increased levels of PARylated proteins (due to suppression of PARG-mediated PAR degrading activity) compared with controls (e.g., non-specific dsRNA) (Figure 2f), but did not influence mycelial growth. Thus, PARylation levels maintained in untreated mycelia presumably reach the threshold that is sufficient to ensure normal mycelial growth.

These data obtained with dsRNA-mediated RNA silencing of the *PiPARP2* gene were confirmed in experiments using the PARP inhibitor 3-aminobenzamide (3AB) [34], which showed that reduction in PAR accumulation (Figure 2g) is associated with decreased *P. infestans* mycelial growth (Figure 2b,e). Thus, the genetic (RNAi) and pharmacological (3AB inhibitor) studies have revealed a positive correlation between the accumulation of PARylated proteins and the mycelial growth of *P. infestans*.

Finally, to perform the virulence assay of *P. infestans* deficient in PAR content in potato leaves, mycelium was collected in sterile water, mixed with or without 3AB and stimulated to release zoospores. To infect potato leaves, zoospore suspension was drop-inoculated on leaves from cv Gala and placed in a dark incubator at 16 °C. At 4 dpi, 3AB-treated leaves did not display any characteristic symptoms of *P. infestans,* apart from minor black holes in the site of inoculation, which may be caused by mechanical wounding compared with control (0.6% DMSO as a solvent for 3AB), whereas leaves infected with *P. infestans* in the absence of 3AB displayed up to 60% damaged leaf surface (Figure 2h,i). At 7 dpi, only initial symptoms of *P. infestans* were observed in the edges of leaves treated with 3AB, whereas 100% of leaves displayed late blight symptoms in the absence of 3AB.

## 4. Discussion

ADP-ribosylation is a dynamic covalent modification of proteins and nucleic acids, which includes the transfer of the ADP-ribose from NAD^+^ to its specific substrates and, meanwhile, releasing nicotinamide. This posttranslational modification has been extensively studied in humans due to its prominent medical impacts on various malignant and inflammatory diseases, including cancers, diabetes and ischemia [5]. Major biological processes such as DNA damage repair, stress responses, immunity, programmed cell death, transcription, chromatin remodelling and metabolism were shown to be modulated by ADP-ribosylation in humans and animals [5,6,14,16]. Plants, like animals, also control multiple cellular processes through the ADP-ribosylation [5,7,8,14]. However, although extensive studies have described cross-talk between ADP-ribosylation and plant responses to biotic and abiotic stresses, the role of ADP-ribosylation remains largely unexplored. Identification of ADP-ribosylated proteins is critical to define the function of ADP-ribosylation in diverse cellular processes. However, only a very few PARP targets have been identified and characterised in plants. Information on ADP-ribosylation in fungi is even more limited: there are only a few reports available in this area. PARP homologue (PrpA) identified in *Aspergillus nidulans* was shown to be an essential protein with a role in the DNA damage response, farnesol-induced cell death and initiation of asexual development [19]. In contrast, *Neurospora crassa* PARP orthologue (NPO) is dispensable for DNA repair or cell survival but may control replicative aging [20]. An excellent study by Gao et al. [22] unveiled a mechanism by which ADP-ribosylation mediated by PARP1 regulates the virulence of *Magnaporthe oryzae* through its involvement in appressorium formation and plant penetration. Furthermore, the authors identified two *M. oryzae* 14-3-3 proteins, GRF1 and GRF2, as substrates of PARP1. Moreover, ADP-ribosylation regulated 14-3-3 dimerisation and was required for the activation of the mitogen-activated protein kinases (MAPKs). Another interesting paper by Wang et al. [21] investigated the functional role of PARP1 in *Fusarium oxysporum* f. sp. *Niveum*. The authors showed that PARP1 contributed to *Fusarium* pathogenicity through interacting with serine/threonine protein kinase, FonKin4, and this interaction was required for vegetative growth, conidiation, macroconidia morphology, abiotic stress response and pathogenicity of the fungus [21]. Thus, functions of ADP-ribosylation in fungi are very diverse and presumably depend on interacting partners.

The role of ADP-ribosylation in oomycete pathogens has not been addressed elsewhere. Here, as the first step to fill this gap in knowledge, we demonstrated that the *P. infestans* genome contains two PARP-like proteins (PiPARP1 and PiPAR2), one of which (PiPARP2) possesses PARylation activity. Using genetic (RNAi) and pharmacological (PARP inhibitor 3AB) approaches, we also found that this PARylation activity presumably plays a crucial functional role in the life cycle of the plant pathogenic oomycete *P. infestans*, being an essential factor for mycelium growth, and possibly may play a part in regulating other stages of its development and capacity to invade host plants. In this brief report, we have not investigated mechanisms underlying ADP-ribosylation activities in *P. infestans,* but, by analogy with other organisms, we believe that ADP-ribosylation may operate through interaction with binding partner proteins and substrates. Thus, identification of the targets, binding partners and metabolism of a once enigmatic protein modification is a priority for future research. One challenge in developing techniques for such investigation (for example, large-scale proteomics) is how to overcome the chemical and structural complexity of ADF-ribosylation.

Future research will also include further genetic evidence generating and using Cas-CRISPR knock-out mutants, which allows us to look at the role of different PiPARPs and PiPARG at different stages of infection in detached leaves as well as in intact plants. We will also need to examine the mechanism of 3AB action in more detail. This PARP inhibitor structurally mimics the nicotinamide part of NAD, and, therefore, effects on other NAD-dependent processes beyond PARylation are likely [34,35,36,37,38]. However, this inhibitor is much more selective than others, such as 3-methoxybenzamid, which may cause a large number of mRNA abundance changes, suggesting pleiotropy and off-target effects of that PARP inhibitor [36]. Thus, in combination with RNAi-based results, the involvement of true PARylation inhibition by 3AB on *P. infestans* seems to be preferable. Moreover, in human and animal systems, 3AB is still widely used as a PARP inhibitor [39]. Nevertheless, we will expand a range of PARP inhibitors with different mechanisms of action to confirm the specificity of 3AB activity.

Taking into account that in many other organisms, including *F. oxysporum* f. sp. *Niveum,* ADP-ribosylation plays a central role in biotic and abiotic stress responses, it would be critically important to analyse the impact of PARP/PARG/PAR deficiency on *P. infestans* growth and development under different stress conditions. In addition, *P. infestans,* like other hemibiotrophic plant pathogens, employ a biphasic infection strategy, initially behaving as biotrophs, where minimal symptoms are exhibited by the plant, and, subsequently, as necrotrophs, feeding on dead plant tissue. The regulation of this transition is not well understood. Given the potential particular role of PARylation in programmed cell death, future research might also provide new insights into the mechanisms underpinning the transition from biotrophy to necrotrophy.

In conclusion, this work opens up a new avenue to identify PARylation patterns and targets in *P. infestans* and also host plants during their interaction. On a practical level, genetic alteration of the ADP-ribosylation process (by affecting *Phytophthora* PARP and PARG proteins) for crop protection management may be achieved by dsRNA-mediated RNAi-based methods. In humans and animals, PARP inhibitors are considered potential tools for the treatment of several serious diseases. A similar strategy could be applied for crop protection. In spite of the certain off-target effects of known PARP/PARG inhibitors, development of the next-generation inhibitors is required to enhance plant stress tolerance resulting in improved growth and yield.

## Data Availability

The *P. infestans* strain VZR ViR21a genome sequencing data are available at BioSample accession SAMN434845453.

## Supplementary Materials

The following supporting information can be downloaded at: https://www.mdpi.com/article/10.3390/jof11010029/s1, Figure S1: sequences of constructs for synthesis of dsRNAs; Figure S2: sequences of *Phytophthora infectans* VZR ViR21a PARP1, PARP2 and PARG genes; Table S1: primers used for quantitative RT-PCR.
