# Supplementary material for: Poly ADP-Ribosylation in a Plant Pathogenic Oomycete Phytophthora infestans: A Key Controller of Growth and Host Plant Colonisation
Source: J Fungi (Basel). 2025 Jan 3;11(1):29. doi: 10.3390/jof11010029 (PMC11766942; doi:10.3390/jof11010029)
Supplement: Supplementary file 1 [file jof-11-00029-s001.zip › jof-3328656-supplementary.pdf]

Supplementary Materials

Article title: Poly ADP-Ribosylation in a Plant Pathogenic Oomycete Phytophthora infestans: A Key Controller of Growth and Host Plant Colonisation

Authors: Viktoriya O. Samarskaya, Sofya Koblova, Tatiana Suprunova, Eugene A. Rogozhin, Nadezhda Spechenkova, Sofiya Yakunina, Andrew J. Love, Natalia O. Kalinina and Michael Taliansky

The following Supporting Information is available for this article:

Figure S1

Sequences of constructs for synthesis of dsRNAs

> dsRNA_ PiPARP2 (nts1225–2037)

CGATTGCCTACCGAGGTGCAGGACTTTTTGACGCTCGTTGCGGACCACACGGAGCTGTCGTATGCTTATA

CAGTCACAGACGAGCTGTTGGGGGACGCTATCATGAAGTTGCCACTCGGCCGTCTGTCCAAGACGACCA

TTAAACGGGGATACGAGACGCTGGAGGAGATTTACGAAGCGCTTATGAGTCGACAGGTGTCTCGAGCG

CGACTGTCGAACCTCAGTAGCCACTTCTACTCACTTATTCCGCACCATTCGCGTCTGGAAGTTATTGATAC

GATGGTGAAACTAGGCAAGAAGGTCCAGCTGATGGCTGAAGTCGGGGCGAGTGTGCGTGGGATGGTT

CACGAGTTTGTGGCTCGACGGTGTGCGTCGAGAAACTACTTGGATGCGTGTTACGATTTGTTAGAGTGT

GAGCTGCAACCTGTGAAGAGCTCGGACCCCGATTTTGCGCTAATTCAGACGTACATTCGTAACTCCAACT

GCTGCGGGCGGAAGCAAGACCGATTGCAGTTGCTGTCGGTCTTTCGCGTTGAGAAGCCCGAGCAGGAG

ACGCGCTTTGAGCCTTTCCGCACATTTACCAACCGACGGATTCTCTGGCATGGCAGCCACTTGGCGAACT

GGTTGGGCATCTTGTCGGAAGGGCTGCGTATCGCACCGCCTGAAGTTGCGTCGAATGGACGGACGTTT

GGTAAAGGACTGTACTTCACGGATAAAGTGACAAAGGCACAGGCGTATTGCCACTGCAGACCTACGAA

CGGCCATAATCAGTGTGTATTCGCGCTGAGTGAAGTGGCGTTGGGTGAGAGCAAG

>dsRNA_ PiPARP1 (nts 1594–2255)

CTCATCTGCGACCCAGAGGTGGTGACGCGAGAAATGGCCAGCCTGAATGTGGACCTGAAGCGCTTTCC

GCTCGGTAAACTGTCGAAGGCGCAGATATCGCAGGGCTACGAAATCCTACAGCGCCTTTCAGCTGCATT

GGAGGAGATGGAACAGCTGGCAACAGCTTCTGCTCCAGCCACGAAAGCCAAAGCTGGCGCGAAAAGTA

GACGAAAGGCCAAGGCGAAAGCGAAGGGACCGACTGCAGCTACCACCAAAAGTCTGTCGGCACTCCAA

GCAGCGATCAAGTCGCTCTCCAGCGAGTTTTACTCGCTCATCCCGCACGATTTCGGTCGCTCTTTGCCGC

CAGCTATCGATACGATGGCAGATCTCAAACTGAAGCTGGAGCTGCTCGAAGTGCTGTCGAATCTGGAGA

TCTCGCAGATGCTGCGGAAGCAAGAAGCCGAGAAACGGAGCGGACCTGCGATCCATCCTCTGGACATG

CACTACAACATGCTCAACACGAACATGGAGCCGCTGAAAAAGCGCGGCAAGGAGTACAAGATCATCGA

GAGATTTATCCAGAAGACCCACGGCGGTAGCAAGCTCAGTATCAACACAATTCTCAAGATTGCGCGTCC

GGACGAAGACACCCACAAGGACGTGCTAGGATCTCTTGACAACCA

> dsRNA_ PiPARG (nts 1–680)

GTTGCTGACGCGAGAGGAACGGGTCGCGTTCTTCAAGGACGTCCTGCCGGAGATGGTGCGACTGGTGC

TGGAAATGCCGCAAATGTTTGCTACTCCGCCGCCACTATTGACGCCTCTGGAGACAGACAAACAGGTGG

AAGGCAAAGCTCCACGTACTGCTAACAGGACTCGAGTGGAGACGCAGACGCATCGGTTTACGAAGCTG

GAGGTGCTGACACTCGTGTGTAGCTGCTTCTTCGGCATTTCTCCTGACCAAGACATCGTCCAATGCGTCC

AACTCGATGGCAGTCCGCAGAGCAAGAAAGCGGCACGCCGCGACAGTGAAGACGACGTCATCCAGTTC

CCCTACTTCACAGCAGTTCGCATGTTCTCAGCTCCTGGAAATATGGGCCGAGTGGTTGTGCTGAAGGCG

CAGAAGATCCGCTGTCTCTTGCAATACTTTTTGCGTGTGGTGCCACGTGCCATCTCCGAGCGTCCAGCGT

TATCGACCGAGGTGATCGACTTTACTCGTGTTGGCGTCCACGTCCCACTTGCCCAAGGTCGACTCAGTGC

TGAGCGAACAACTCAAGAACTGCTCACGATATTGTCGACTAGTGAGAGTAATACTGGTGCTACCGCGCA

GATATGTCCCCCTCACTTACACGCTGCTCGATGCGTCTCGGACACGTTGATCGAAAATC

Figure S2

Gene sequences

P. infestans strain VZR ViR21a

> PiPARG (partial sequence)

GTTGCTGACGCGAGAGGAACGGGTCGCGTTCTTCAAGGACGTCCTGCCGGAGATGGTGCGACTGGTGC

TGGAAATGCCGCAAATGTTTGCTACTCCGCCGCCACTATTGACGCCTCTGGAGACAGACAAACAGGTGG

AAGGCAAAGCTCCACGTACTGCTAACAGGACTCGAGTGGAGACGCAGACGCATCGGTTTACGAAGCTG

GAGGTGCTGACACTCGTGTGTAGCTGCTTCTTCGGCATTTCTCCTGACCAAGACATCGTCCAATGCGTCC

AACTCGATGGCAGTCCGCAGAGCAAGAAAGCGGCACGCCGCGACAGTGAAGACGACGTCATCCAGTTC

CCCTACTTCACAGCAGTTCGCATGTTCTCAGCTCCTGGAAATATGGGCCGAGTGGTTGTGCTGAAGGCG

CAGAAGATCCGCTGTCTCTTGCAATACTTTTTGCGTGTGGTGCCACGTGCCATCTCCGAGCGTCCAGCGT

TATCGACCGAGGTGATCGACTTTACTCGTGTTGGCGTCCACGTCCCACTTGCCCAAGGTCGACTCAGTGC

TGAGCGAACAACTCAAGAACTGCTCACGATATTGTCGACTAGTGAGAGTAATACTGGTGCTACCGCGCA

GATATGTCCCCCTCACTTACACGCTGCTCGATGCGTCTCGGACACGTTGATCGAAAATCTGGACAGTCAT

CTGCAGG

>PiPARP 2 (full sequence)

ATGAATCAGTGCATACAAACATCAAAACTAAACGACTCCGACCCGGCCCAGGAGCACAGCAGACCCGCC

ACCGCCGAAGAAGGGAGGAAGCCGCAGCCCCAGTTCAGTTCCAGCCGCCGGAGCCGCAGACAGATAG

ATACACTCAAGCGACGGATCGCCACGATGGCCAAGAAGCGTCGCGCGTCCACATCTTCTGCCCAGAAGG

CGTCGCCTGCCGCTGCTGCCATGATCTCGTCGCCCTTCCCGTACCCTTCTCCGGCGACGCCCAGTCGCTC

GAGTCGCTTCAACTTGCCGCGCGTACACACGTCGCGTTCGGGTGTGGCCGCCAGCACGCGCTCGCAGCT

CACTGTGGCGCAGCTGCCTGTATTGTCACCTCCACTCAAGAAACGCGTCACGTCGCGACGCAGCGCCAA

AGCCGTCAAACTCCAGTTGCCAGACGACGACGTAGATGAAGACGAGAATGATGGTAGGACCAACTGCT

GCGTGCACAACAGCGACACCGAGACGGAGGACGATGAAGAGAAGCTGCTGCTTATTAAACCTGATCCG

TATCTGCCTGAGTCCTATCAACACCGAGCCGCCGTACTCCGACACGCTGGGAGCTTCTACGACGCCATGC

TCACTCGAGCGCGTGTCCACAACAACCACAGCGAGTTCATCGCGTTACAGGCGCTCGAGTTCGACAAGC

AGTTCTATCTCTGGACCAGAGCTGGACGTGTCGGATGTGCAGGCAAGACGATGCTCACAGGCCCTTTCC

CGAGTGCTGAGAAGGTCACACTCGAGTTCTGCACGTTGTTCGCATCCAATACCAACTGCGAGTGGGAGG

AGCGTCATGCTCTGAGATACCAAGAAGGCAGCTACACGTGGATCGAGCTCGACTATTCAACTGGAACGC

CGTATTCCGAGTCGCTAACATCGGCGCTGGCAGACAATCAGGGAGAAATGGACAACGCAGACAGTTTC

CGAGACTTTGACCTGGTTCCGACGGCGTCTCCGCCGCTTCCGATGGCTCCGTCTAATACGCGCAAGCGAC

GTCGAGCGGGTATATGGACGACGTACTCCACTGCAACGTCAACTTCGACCACGACTGCAACGTCCAGCA

CCAGTGTACGACGGTCGCTGAGCAACCAGTTCGACTGGCTGTATGCAGGTTCAGACGGAGACACGGCC

AGTGCAGCGTTCACCGAGACTTCCACGTGCGCGTGTGCCACTGCGCCGCCGTCGCGATTGCCTACCGAG

GTGCAGGACTTTTTGACGCTCGTTGCGGACCACACGGAGCTGTCGTATGCTTATACAGTCACAGACGAG

CTGTTGGGGGACGCTATCATGAAGTTGCCACTCGGCCGTCTGTCCAAGACGACCATTAAACGGGGATAC

GAGACGCTGGAGGAGATTTACGAAGCGCTTATGAGTCGACAGGTGTCTCGAGCGCGACTGTCGAACCT

CAGTAGCCACTTCTACTCACTTATTCCGCACCATTCGCGTCTGGAAGTTATTGATACGATGGTGAAACTA

GGCAAGAAGGTCCAGCTGATGGCTGAAGTCGGGGCGAGTGTGCGTGGGATGGTTCACGAGTTTGTGG

CTCGACGGTGTGCGTCGAGAAACTACTTGGATGCGTGTTACGATTTGTTAGAGTGTGAGCTGCAACCTG

TGAAGAGCTCGGACCCCGATTTTGCGCTAATTCAGACGTACATTCGTAACTCCAACTGCTGCGGGCGGA

AGCAAGACCGATTGCAGTTGCTGTCGGTCTTTCGCGTTGAGAAGCCCGAGCAGGAGACGCGCTTTGAGC

CTTTCCGCACATTTACCAACCGACGGATTCTCTGGCATGGCAGCCACTTGGCGAACTGGTTGGGCATCTT

GTCGGAAGGGCTGCGTATCGCACCGCCTGAAGTTGCGTCGAATGGACGGACGTTTGGTAAAGGACTGT

ACTTCACGGATAAAGTGACAAAGGCACAGGCGTATTGCCACTGCAGACCTACGAACGGCCATAATCAGT

GTGTATTCGCGCTGAGTGAAGTGGCGTTGGGTGAGAGCAAGGAGATGCTCAACTCGGACGACAATGCC

AAGCAGTTTGTGCACACAGGAGCTGGAGGGCGTGCGAAGGGAGCCTACTATCACAGCTGCAAAGGTGT

GGGGTCGTGTCGTCCGGACTCTGCTGGTGAAGTTGTGGATATCCACGGAGCCATTTGGCCCGTAGGGA

AACCAGTCCAACCGGAGGAGCGAACGGGACTGCACCACAGCGAGTATATCATCTACAACCCCAGCCAG

ACTCGGATGCGCTACGTCGTGCTGGCAAGGAGTCCGTACTAA

> PiPARP 1 (full sequence)

ATGGCTCCTCAACAACAAGACACCCAAAGCGCGGGTACGAGCACCACTGCGTCTTCCATCGCGGAGAAG

TCGGAGTCTGTTACTAAGCATAAGCCCATCAAGAACGCGCTCAATGATGAGGTCGAGCATTTCGAGTTC

GACCACCCTGAACTTCGCAGCGTTTTCCCCACAACCAAGCACGACCCAATTCCAGTGCTGCCGTACGAGG

ACCGAGGACTGATGGCCGACTTGACGTTCAAGAATTTGCTCAAGGATGCCTCGGTGACGCACTTGTCGC

CAAGGATCGGCACGGAACTGTCGGGCATTCAACTGCATCAGTTGACGAACGCGCAGCGCGATGAGCTT

GCTCTACTGGTGTCACACCGCGGCGTGATCTTTTTCCGTGAACAGGAGATTAATATTGAGCAGCAGCTCG

ATCTTGGTCGGTACTACGGCCCACTGCACGTGCACCAGAATCTCGGTCACCCGGAAGGGCACCCTGTAG

TGCTGGTGGTCGAGAACTCGGTGGGGGACAGTGACCGGATCATCAAACGCCAGCAGTACGACCCAGAC

AACGTGTGGCACAGCGACGTTTCCAACGAGCGCCAACCTCCTTCGTACACAAGCTTCAAAGTGCTAACG

AACCCTCCACTTGGAGGTGGCACGTTGTGGGCGTCCGCATACGAGGCTTACGAGCGCCTCACACCGCCG

TTTAAGACATTCATCGAGGGATTGACGGCGATTCACAGCAGCAAGGCCCAAGCCGAGCGAGCAGGGAG

ACGTGGACACACGATTCGTCGAGCTCCGGTGGAGTTCGAGCACCCGGTTGTACGCACCCACCCGGTAAC

TGGTCGTAAGGCTCTATTTGTCAACCCCGCGTTCACCAGACGCATCCCGCAGCTCTCTTCTCGCGAGTCA

GATGCGGTGCTCAAAGTTTTGTACAAGCACATCACGGAGGGCCACGAGTTCCAAGTTCGATTCCGCTGG

ACCAAAAATGCTGCTGCGGTCTGGGATAATCACATCACGACTCACTTCGCGACGTTCGACTACTTACCAG

GCAACCGACACGCGGTTCGAGTGACTACGCAAAAAGAGATCCCCTACTTGGACGAAAGACCTTTCTCGT

CATTCTTCAATTCGCATCCAATGGCGTCAACTGCCACGATCGTCGACCCGCAAAGCTGCTGCAGCGCCAA

TGCCGAGCTCTATCGCGACGGGGATGGCATGCGCTGGAGCTTCATGTTGAACTTAACGGACATTTCCTA

CGGCACCTACGGCAACAACAAGTTCTACATGGGGCAGCTCATCGTAGACCGCGGGCGCTTCGTGGTCTT

CCGCAAATGGGGCCGCGTGGGCGCCAAGACACCGCAGTCGAAAACTGAGTTCTACAGCAGCGTCGAAG

AGGCTGAGTGGGCCTTCCAGAAGGTCTTCCAGTCGAAAAGCGGCAACAAGTGGCCGCTCACGGAGCCT

TTTGTCCGCAAGAAAGGCAAATACTTCCTCGTCGAGCTGGACGACGGAGAGCCCGAAGGATCATCTGCA

GATGTTGAGGCGAAGGTGGAGGAAAAGCACGCAGTGGCGTCCAAACTGCCACGAGAAGTGCAAAGCA

TCGTCCAGCTCATCTGCGACCCAGAGGTGGTGACGCGAGAAATGGCCAGCCTGAATGTGGACCTGAAG

CGCTTTCCGCTCGGTAAACTGTCGAAGGCGCAGATATCGCAGGGCTACGAAATCCTACAGCGCCTTTCA

GCTGCATTGGAGGAGATGGAACAGCTGGCAACAGCTTCTGCTCCAGCCACGAAAGCCAAAGCTGGCGC

GAAAAGTAGACGAAAGGCCAAGGCGAAAGCGAAGGGACCGACTGCAGCTACCACCAAAAGTCTGTCG

GCACTCCAAGCAGCGATCAAGTCGCTCTCCAGCGAGTTTTACTCGCTCATCCCGCACGATTTCGGTCGCT

CTTTGCCGCCAGCTATCGATACGATGGCAGATCTCAAACTGAAGCTGGAGCTGCTCGAAGTGCTGTCGA

ATCTGGAGATCTCGCAGATGCTGCGGAAGCAAGAAGCCGAGAAACGGAGCGGACCTGCGATCCATCCT

CTGGACATGCACTACAACATGCTCAACACGAACATGGAGCCGCTGAAAAAGCGCGGCAAGGAGTACAA

GATCATCGAGAGATTTATCCAGAAGACCCACGGCGGTAGCAAGCTCAGTATCAACACAATTCTCAAGAT

TGCGCGTCCGGACGAAGACACCCACAAGGACGTGCTAGGATCTCTTGACAACCACATGTTGCTGTGGCA

CGGCTCCCGTCTGTCGAATTTCGTGGGCATTCTATCGCAGGGGCTGCGTATTGCCCCGCCTGAAGCTCCC

AAGAACGGATATCAGTTCGGCAAGGGGGCAGTGCTGCTGCTCGCAGACGTCGCACTGGGCACGCCGTT

CAAGACTCCAAACGGCGAGTTTCTGGACTACAAGACGGTGAAAGAGCAACGCGGATGCGACAGCACAC

ACGGACTTGGACGCATGGCACCGGCGGAAAATGAGTTCGAGACGCTGCCCGACGGCGTGGTTGTGCCC

GCCGGTACGCTGAAGGCAGTGGACGGTAACCAGTATTTGATGTACAACGAGTTCATCGTCTATCGCCGC

GAGCAGGTCCAACTGCGCTATCTGGACGCGAATGGTGTCGCCTGGAGCTTCATGCTCAACTACACCAAC

ATCTCGTTCGGGACGTATGGAAACAACAAATTCTACATGGTGCAGCTGATCCAGGTCGGCAATAACTTC

ATGGTCTTCCGCAAGTGGGGACGCGTCGGAGCCAAAAACCCGCAGCGCGCTCTGGAGCGCTACAACAC

GTCGCTGGAGAAAGCGCAGGCGTCTTTTACGAAAAAGTTCTTGGACAAGAGCGGTAATGCATGGCCATT

GACAGGGCCGTTTGAGAAGGTGGAGGGCAAATACGTGCTCGTTGAACTGGACGACGAAGTGGCAGAA

GAGGAGGAACAGATGAGCGACGTCGAGAAGGAAGAAGAGGTGGCGTCGACTCTACACGAGACTG

TCCAAGAAGTGTTGAAGATCTCGCGGGGTTACGCGTTGCTCCAGCAGCTCTCTGAAGTGGTCAAGGAAA

TTGAAGATCTCAACAAGGTTGCTGCAAATACTCATGAGGCACCTCCTGCAAAGCGGACCGGAACACGAA

GATCCACTCGGGTCAAACGAGCAGCCAAGCCAAATGCCGTGCAGATCCGCCGCTTAAAGGCTGGTCTGA

AGACCCTTTCAAGCGAATTTTACACTCTGATTCCGCACGACTTCGGGCGAAACTTACCGCCCCCGATCGA

TTCGCTGGACGAGGTGAAGCTGAAGAGTGACTTGCTCGAGGTGCTGGCGAATATCGAGATTTCGCAGA

AGTTGCAGGCTGAAAAGAAAAAGAATGCAAAGAAGAATGCCGGCGCTAAGCTGAACTCGCTCGACGCG

CAGTACAACATGCTTAACGTCAAGATGGAGCCACTTCCTGAAGCTACCGAGGAATTCAGGATCATCGAA

AAATACGTGGAGACCACGCACGCTCCGACCCACGTGCAGTATAAACTGCGGATAAAATCTGTTCTCAAG

ATTGCGCGTCCGGATGAAGAAAAGTTCAAGGACGTTTTTCAGTCCGTGAACAACCACAAGTTGTTGTGG

CACGGGTCTCGCCTCTCGAACGTCATCGGTATCTTGTCTAAGGGCCTTCGCGTAGCCCCACCCGAGGCAC

CAAACAACGGATACATGTTCGGCAAAGGAGTGCGTGGAGATTACTTCACTGAGGTGGCTTTGGGTGCCC

CCTACAAGGCTCAAGAAGCGGACGATCTGACGTATACAACCTTGAAGAAAACCAAGGGATGTGATAGT

ACACACGGAGTTGGCCGCATGTCTGCCCTCGAAGAAGACTACGAGACCATGGAGGACGATGTGGTTGT

TCCCATCGGTGAGTTAATGCCATCGGACGGCAGTGGCTCGTTGCTCTACAACGAGTTTATCGTGTACCGC

CAGGAACAAGTGAAGTTGCGGTATCTGGTCAATCTCGACTTCCTATTCGAGGACGAGGAAGAGGAGGA

GGCGTAG

Table S1 Primers used for quantitative RT-PCR

Primer	5'-3' sequence	Name	Primer concentration (nM)	E (%)
PITG_04640-F<br>PITG_04640-R	GGCCCCGTTGGTTTGTC<br>GCAGAGACTCGTGGTGCATCT	*P. infestans* ef1a elongation factor	300	91.2
PITG_11766-F<br>PITG_11766-R	TCTCGCGCAAGGACTGGTA<br>CAGTTGCGCTCCGAGAAGAT	*P. infestans* 40S ribosomal protein S3a	350	97
dsPARP1-F<br>dsPARP1-R	TCGGAGTCTGTTACTAAGCATA<br>CTACAGGGTGCCCTTCCGGGTGA	dsPiPARP1	290	90.3
PARP2-F<br>PARP2-R	CACTGTGGCGCAGCTGCCTGT<br>GCGATGAACTCGCTGTGGTT	dsPiPARP2	300	95
dsPARG-F<br>dsPARG-R	TAGCTGCTTCTTCGGCATTTCTC<br>CCTGCAGATGACTGTCCAGA	dsPiPARG	320	96
